# Bilharzia Disease

**Published:** 1902-05-17

**Authors:** 


### BILHARZIA DISEASE.

A REMARK was made by Surgeon-General Keogh at a recent meeting of the Medical Society, which should not go entirely without notice. Commenting upon a paper by Lieutenant Lelean on the Bilharzia Hæmatobia he expressed the opinion that this disease would shortly become of much importance in this country, for it was not unlikely that cases would occur, and that they would be mistaken for hæmaturia from other causes or for chronic dysentery. The subject becomes of importance to us at the present time in consequence of the prevalence of Bilharzia disease in certain parts of South Africa, and the certainty that out of the large number of troops now engaged there a considerable number of men will be invalided home while suffering from it. The Bilharzia parasite is common in many parts of Africa. It has proved a great pest to the French in the Algerian hinterland, and it especially prevails on the eastern side of our South African possessions. Cases have been invalided home from the Rustenberg district, from the Swaziland border, from the Zambesi and from the Crocodile River. Many of them seem to have been attributed to bathing, but the exact mode in which the parasite gains access cannot be said as yet to be accurately known. The important thing is that these cases must be looked out for, the urine especially being carefully examined for the ova which are often discharged by that route in very large numbers, either free or entangled in the casts and blood clots which are found in these cases.

